# 4D flow jet shear layer detection method for the measurement of effective orifice area and assessment of aortic stenosis severity

**DOI:** 10.1186/1532-429X-15-S1-P241

**Published:** 2013-01-30

**Authors:** Julio Garcia, Alex J Barker, Susanne Schnell, Pegah Entezari, Riti J Mahadevia, Philippe Pibarot, James Carr, Michael Markl

**Affiliations:** 1 Radiology, Northwestern University, Chicago, IL, USA; 2 Biomedical Engineering, Northwestern Universi, Chicago, IL, USA; 3 Medicine, Laval University, Quebec, QC, Canada

## Background

Aortic stenosis (AS) is the most common cause of valve replacement and its severity is mainly assessed by transthoracic Doppler echocardiography (TTE) to quantify valve effective orifice area (EOA) as determined by the continuity equation. In a previous study we demonstrated that EOA can be directly determined with 2D flow MRI downstream of the AS using the jet shear layer detection (JSLD) method and the acoustical source term (AST) concept. However, both TTE and 2D flow MRI rely on the measurement of local and single-directional velocities and thus incomplete assessment of the complex post-valve flow dynamics in a significant proportion of patients. 3D CINE phase contrast MRI with 3-directional velocity encoding (4D flow MRI) may improve EOA estimation by the JSLD method coupled with full volumetric coverage of ascending aortic 3D blood flow. The objective of this study was to validate 4D flow MRI based EOA estimation using an in-vitro stenosis phantom and to apply the technique for the in-vivo measurement of the valve EOA compared to the reference standard 2D flow MRI.

## Methods

In-vitro test consisted of a glass stenosis model (pipe Ø 33.5±2.0mm, stenosis Ø 10±1.0mm, EOA= 0.78cm2) filled with a blood-mimicking fluid and measured at steady flow (5.7±0.5L/min). In-vivo study included 35 subjects: 10 healthy control subjects (60% men, age 39±11 years) and 25 patients (76% men, age 44±11 years) with mild to severe bicuspid AS (0.95cm2≤EOA≤4.56cm2). 4D flow MRI was performed at 1.5T and 3T system (Avanto, Skyra, Siemens, Germany). Data was acquired in a sagittal oblique slab covering the entire aorta (spatial resolution/temporal resolution=2.5×2.1×3.2mm3/40-50ms). As reference standard, EOA was calculated using continuity equation (EOA-CE=SV/VTIAo, where SV is the LV stroke volume and VTIAo is the aortic velocity-time integral using 2D flow MRI). The JSLD method (EOA-JSLD) was employed to calculate EOA from 4D flow data by using AST([▽(ωΛV)], where ω is vorticity and V is velocity field) to detect the post-valve jet-flow zone, i.e. EOA.

## Results

**Figure 1:**
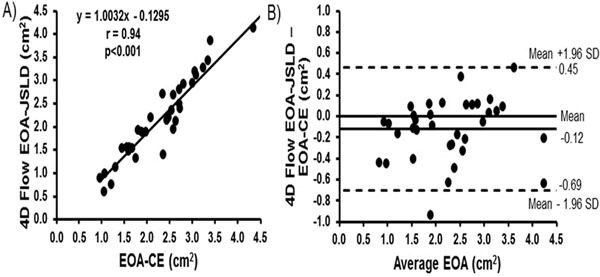
Regression and agreement of valve effective orifice area methods. Panel A shows the regression fit of the valve effective orifice area (EOA) measured by the continuity equation using 2D flow MRI (EOA-CE) and the 4D flow MRI EOA measured by the jet shear layer detection (JSLD) method (EOA-JSLD). Panel B shows the corresponding Bland-Altman agreement plot for both methods.

**Figure 2:**
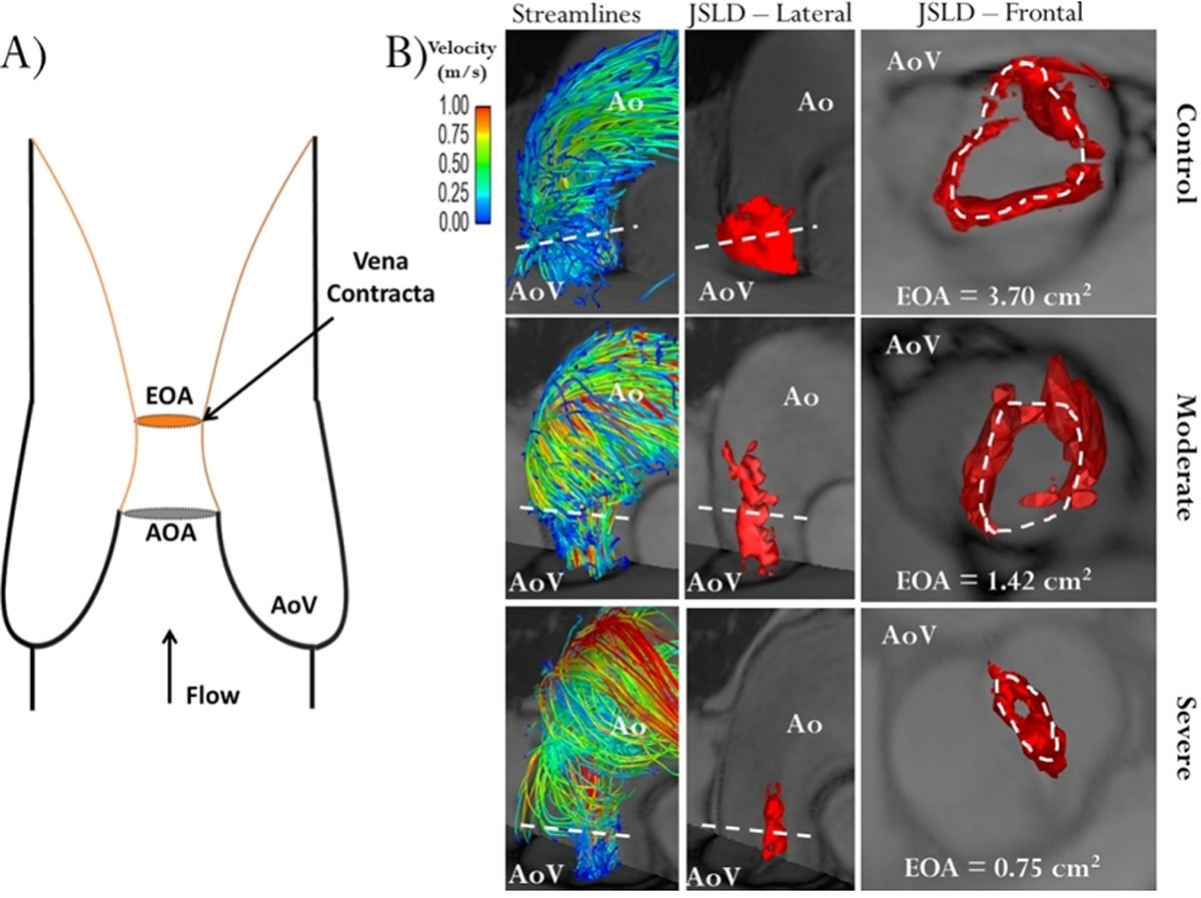
Examples of valve effective orifice area estimation. Panel A shows an idealized representation of transaortic valve flow separation. AOA is the anatomic orifice area and EOA is the valve effective orifice area at the vena contracta (smaller area of transvalvular flow reattachment, orange lines, and maximal velocity position), note AOA>EOA. Panel B shows three different cases (control, moderate and severe aortic stenosis) using valve area estimation with the 4D flow jet shear layer detection method. The first column illustrates the aortic flow velocity streamlines at peak systole; the second column shows a 3D lateral view of the acoustical source term (AST) structure, red iso-surface, computed from 4D flow MRI data at peak systole; the third column shows a 3D frontal view of AST at peak systole. Dashed white line indicates transvalvular maximal velocity position, i.e. the vena contracta.

In-vitro test led to excellent agreement between 4D flow derived EOA-JSLD=0.781±0.018cm2 vs. theoretical EOA=0.785cm2 obtained by the potential flow theory. The determination of valve EOA-CE using 2D flow MRI was 2.07±0.83cm2 vs. 1.90±0.83cm2 (r=0.94, p<0.001) for the 4D flow JSLD method. A Bland-Altman analysis between the EOA-CE and EOA-JSLD methods led to a small mean difference of -0.12±0.58cm2, demonstrating good agreement (limits from 0.45 to -0.69cm2, Fig.1). EOA-JSLD examples are shown in Fig 2.

## Conclusions

This study showed that valve EOA calculated using JSLD method can be easily obtained from 4D flow MRI measurements in AS patients and is in excellent agreement with standard techniques. This method may be useful for accurately grading the AS severity non-invasively without the use of SV or VTIs and thus minimizing measurement variability.

## Funding

Grant support by NIH R01HL115828, NUCATS Dixon Award.

